# Supplementary material for: Chromothripsis during telomere crisis is independent of NHEJ, and consistent with a replicative origin
Source: Genome Res. 2019 May;29(5):737–49. doi: 10.1101/gr.240705.118 (PMC6499312; doi:10.1101/gr.240705.118)
Supplement: Supplemental Material [file supp_gr.240705.118_Supplemental_file_1.zip › contigs/annotated_contigs/DB55/contig.3.DB55_length_594_mean_cov_6.44107744108.docx]

**DB55_length_594_mean_cov_6.44107744108**

```
TTTTTTTTTCTTTCTTTCTTTCTTTCTTTCTTTCTTTCTTTCTTTCTTTCTTTCTTTCTTTCTTTCTTTCTTTCTTT|TTTTGAGACGG
 >chrX:105730289-105730366 + E=1e-31                                           >chr12:13
AGTCTCACTCTGTCGCCCAGGCTGGAGTGCATTGGTGTG|GATTCCGGTGATCGCAGTATCCGTAACAAGAGAACATGCC|ATTCTCCT
2884373-132884423 - E=2e-18 p=6e-03                                             >chr7:5
GAATCAGCCTCCTGAGTAGCTGGGACTACAGGCACCCGCCACCACGCCAGGCTAATTTTTTGTATATTTACTAGAGACGGGGTTTCATC
3868791-53869219 - E=2e-163
ATGTTGGCCAGGATGGTCTCAATCTCTTGACCTCGTGATCCACCCACCTCGGCCTCCCAAAGTGCTGGGATTACAGGCATGAGCCACCG

TGCCCAGCCAGTAGTATTTTTATAACAAAACATTTGATATTTTTTATACTTATTAGGGCTTAGGTCAGATCTACATACTCTGAAAGTCG

GTCAAAGGAAGTTCATTCACATGTGAAAATAAAAGGCACAGGTCTGTCCCAATGTGGTCATGCTGCTTTAACAAAATACCATAGACTGG

GTAACTTAGAAACAACAGGAATTTGTTTCTCACAGTTTAGGAGGTTGGGAAATCCAAGGTTAA
```
